# Supplementary material for: A multimodal dataset for precision oncology in head and neck cancer
Source: Nat Commun. 2025 Aug 4;16:7163. doi: 10.1038/s41467-025-62386-6 (PMC12322140; doi:10.1038/s41467-025-62386-6)
Supplement: Supplementary file 1 — Supplementary Information [file 41467_2025_62386_MOESM1_ESM.pdf]

A multimodal dataset for precision oncology in head and neck cancer

Supplementary Figures

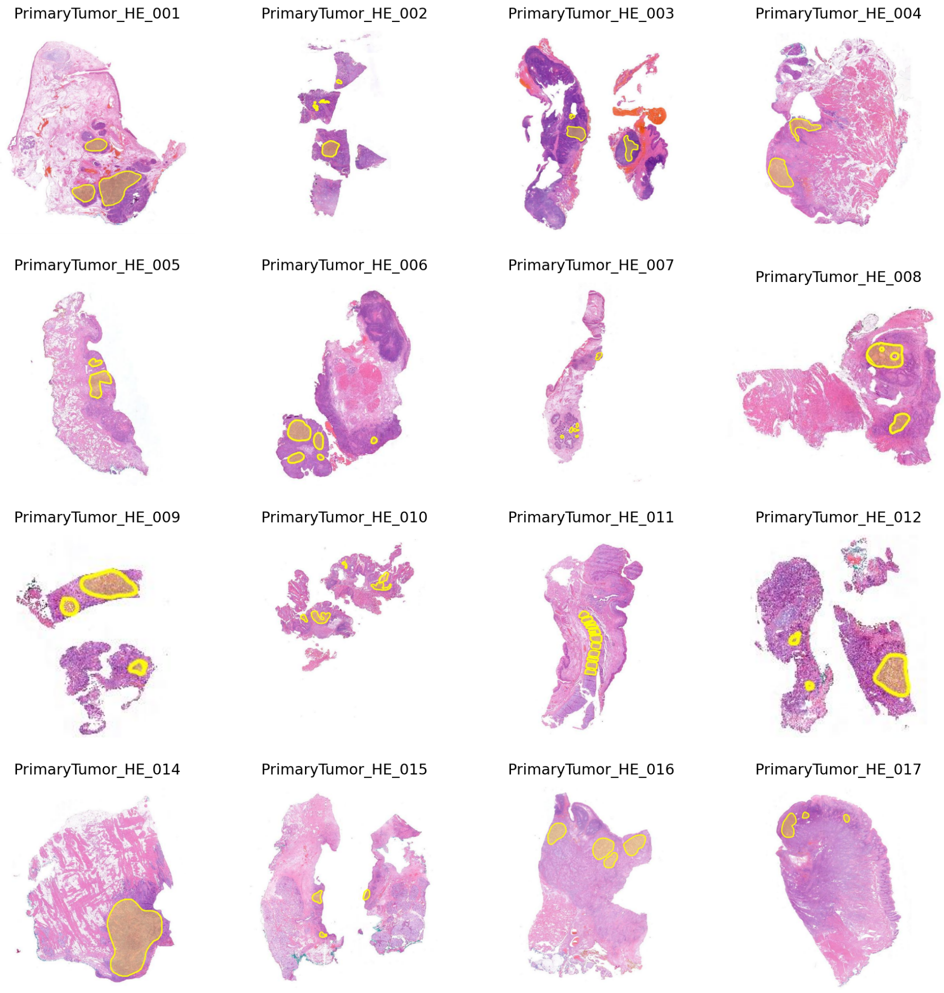

Figure S1 Whole Slide Images of the primary tumor from 16 patients. Sparse manual annotations by trained personnel of representative tumor regions are highlighted in yellow.

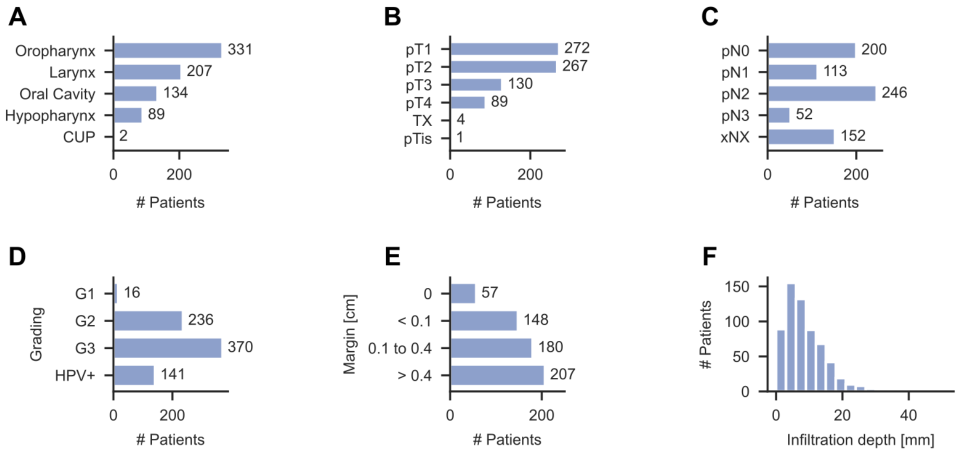

Figure S2 Summary of pathologic data. (A) Number of cases per primary tumor site. There were two cases with cancer of unknown primary (CUP). (B) Cases per pathological T stage, where more specific stages such as pT1a, pT1b were grouped to pT1. (C) Cases per pathological N stage, where more specific stages such as pN2a, pN2b were grouped to pN2. (D) Number of cases vs. grading, where HPV-associated carcinomas were not graded. (E) Distance to closest resection margin, divided into four groups. (F) Histogram showing the distribution of tumor infiltration depth.

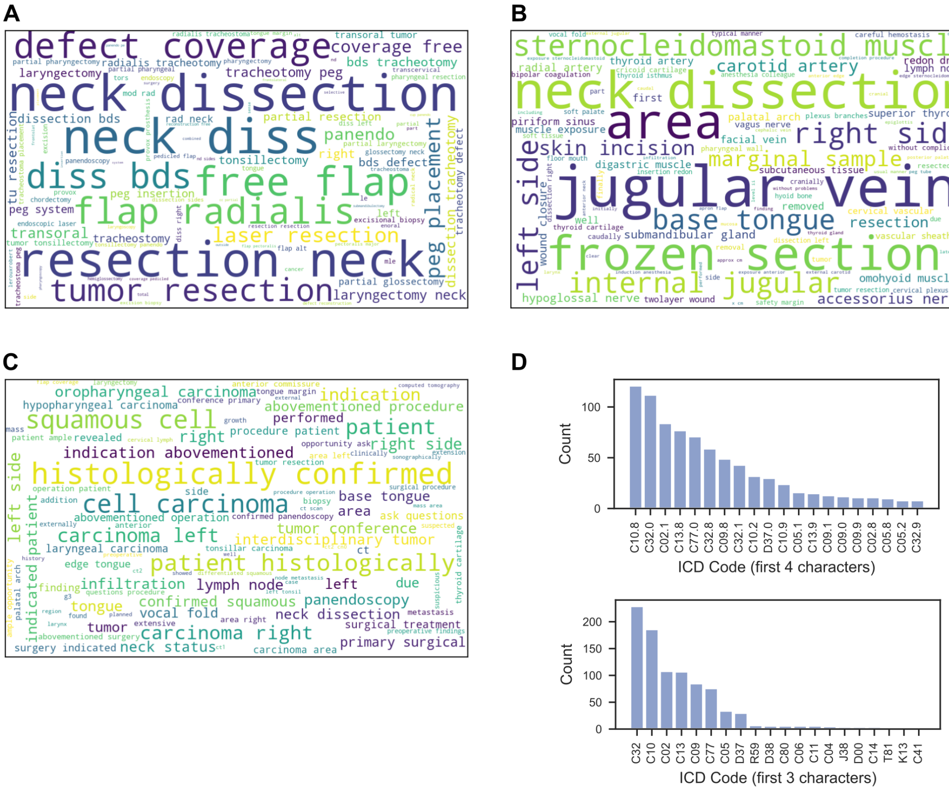

Figure S3 Visualization of text data translated to English and frequency of ICD codes (International Statistical Classification of Diseases and Related Health Problems) of the German version ICD-10-GM. (A) Wordcloud of most frequent terms in short descriptions of surgeries. (B) Wordcloud of the most frequent terms in the surgery reports. (C) Wordcloud of the most frequent terms in medical histories. (D) Frequency of the top 20 ICD codes, considering the first 4 or 3 characters.

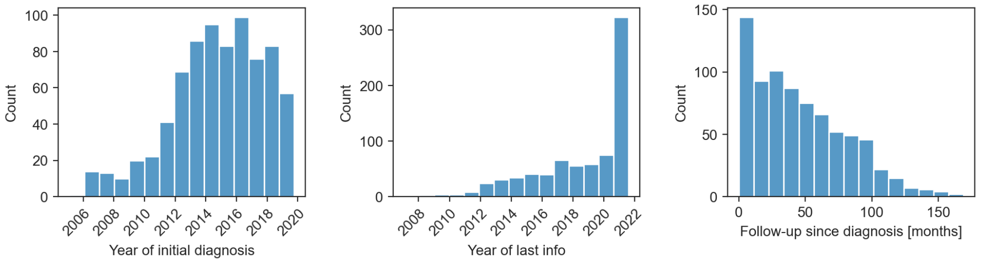

Figure S4 Histograms showing how many patients were diagnosed between 2006 and 2020 along with the years in which the follow-ups ended, and the follow-up time from diagnosis to last information.

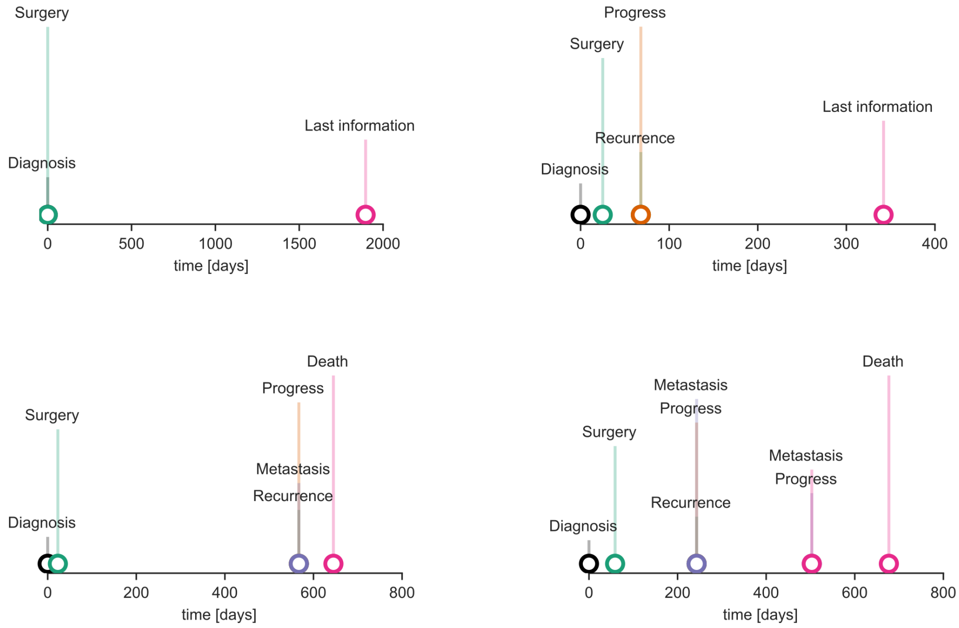

Figure S5 Event timelines of four patients. In the top left example, the diagnosis was histologically confirmed externally, so the unknown diagnosis date was set to the surgery date. Considered events were diagnosis, surgery, progress, recurrence, metastasis, and death. Timestamps of possible adjuvant treatments were not included in the dataset as they were not available for all patients.

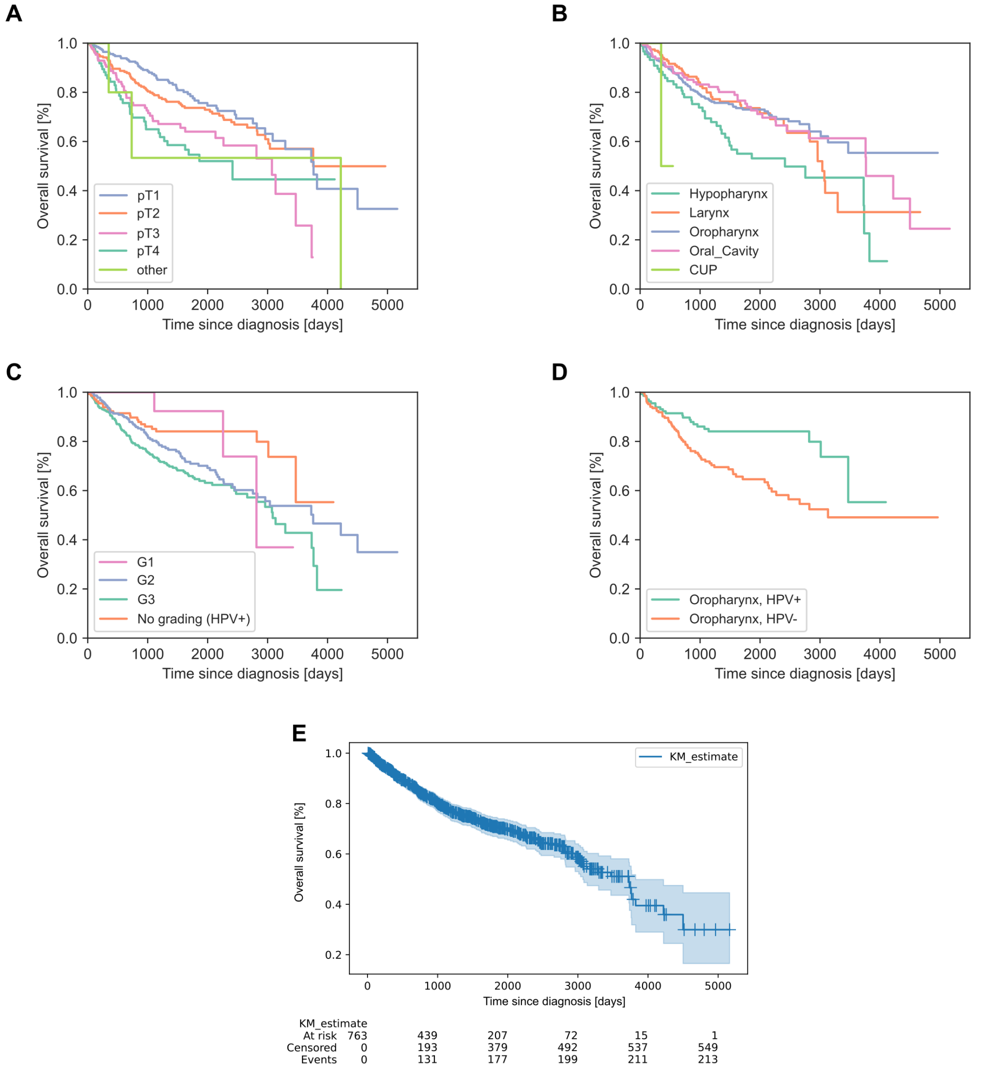

Figure S6 Kaplan-Meier curves of overall survival. (A) Overall survival of patients grouped by pathological T stage. (B) Overall survival of patients grouped by grading. HPV-associated carcinomas were assigned to a separate group as these were not graded. (C) Overall survival of patients grouped by primary tumor site. The two cases with cancer of unknown primary are denoted as CUP. (D) Overall survival of patients with oropharyngeal carcinoma, grouped by HPV status (p16). (E) Overall survival with censored patients (+), the confidence interval, and the number of patients censored and at risk at different time points.

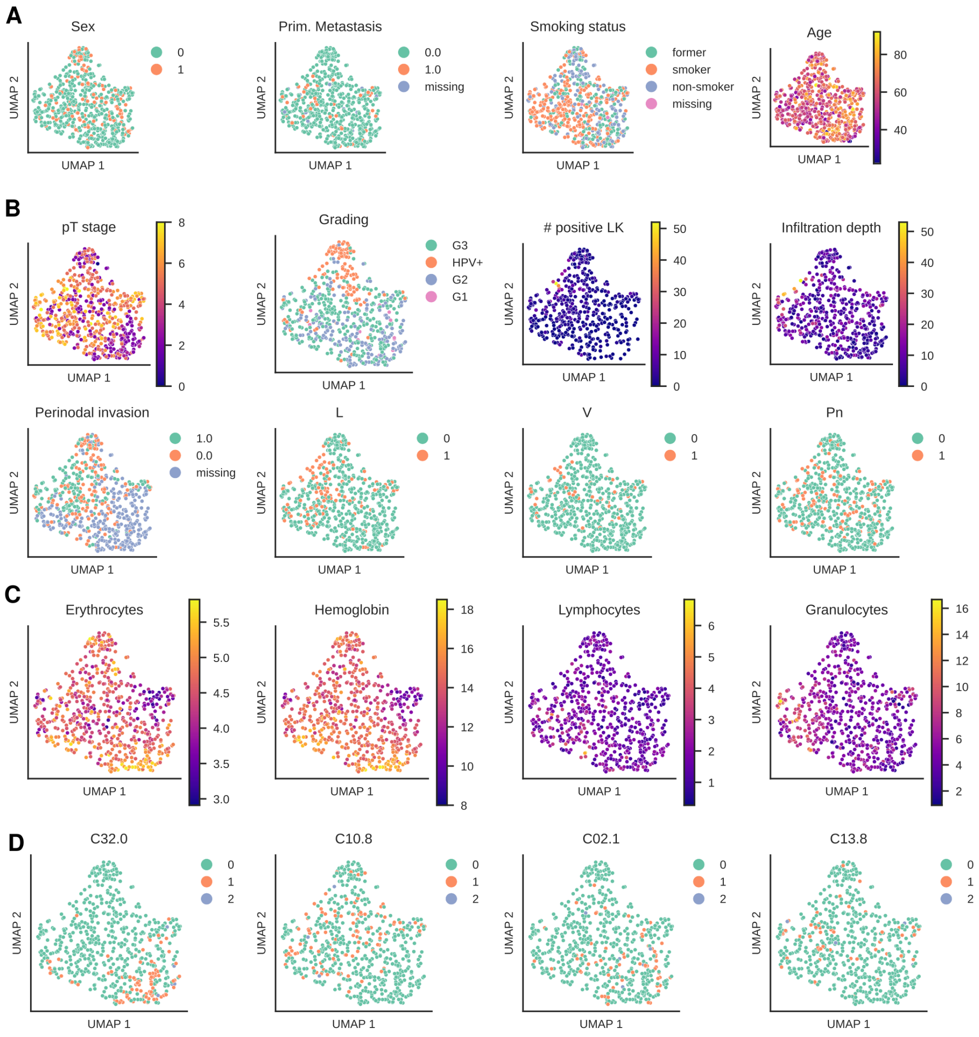

Figure S7 Two-dimensional representation of the multimodal patient vectors, colored by different patient characteristics. (A) Demographics. (B) Pathology. (C) Blood parameters. (D) ICD Codes.

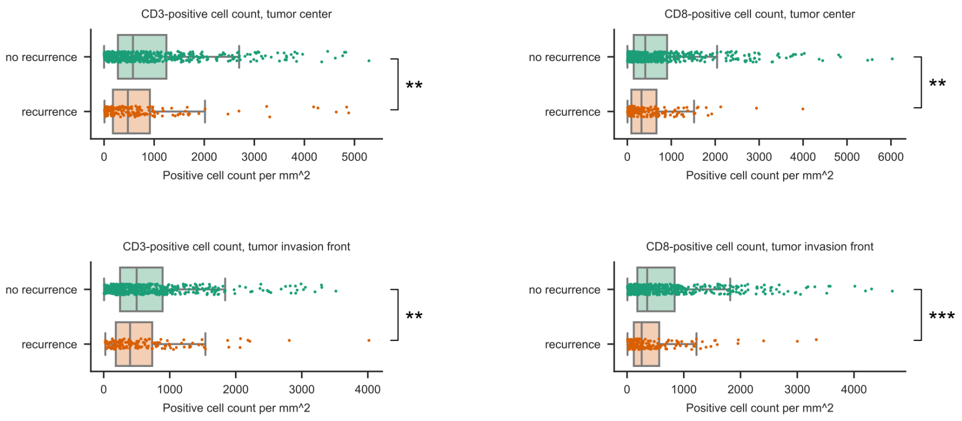

Figure S8 The distribution of CD3- and CD8-positive cell counts per mm^2 were significantly greater for patients with no recurrence than for patients with recurrence. This applied for both the tumor center and tumor invasion front. We used the Wilcoxon-Mann-Whitney test. Two asterisks (**) denote a p-value below 0.01 and three (***) denote a p-value below 0.001.

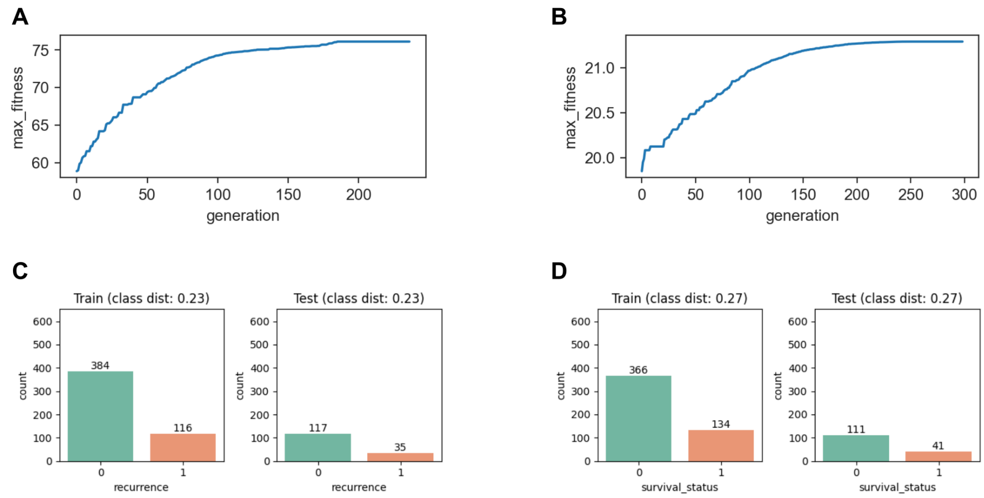

Figure S9 Fitness and class distributions of the genetic algorithm. (A) Maximum fitness for each generation of the genetic algorithm for the in-distribution dataset split. (B) Maximum fitness for each generation of the genetic algorithm for the out-of-distribution dataset split. (C) Distribution of the target class ”recurrence” of the training and test dataset at the last generation. (D) Distribution of the target class ”survival status” at the last generation.

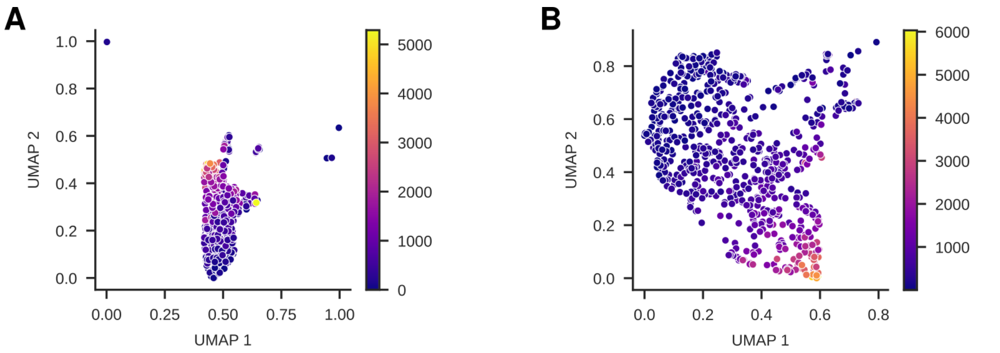

Figure S10 Visualization of image features and cell densities using UMAP. (A) Two-dimensional representation of features extracted from CD3-stained TMAs, colored by calculated CD3-positive cell density. (B) Two-dimensional representation of features extracted from CD8-stained TMAs, colored by calculated CD8-positive cell density.

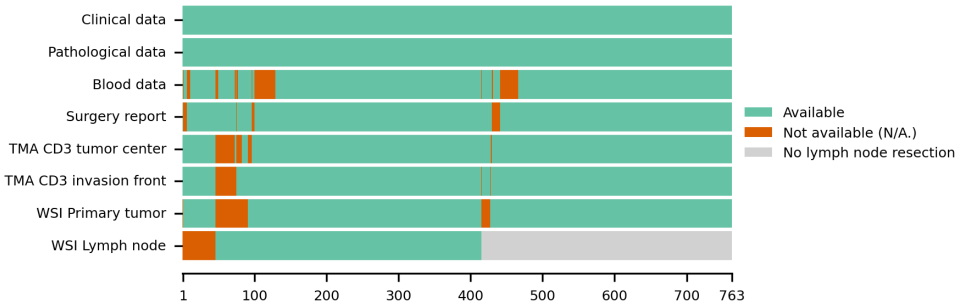

Figure S11 Visualization of the available data. The red areas show for how many patients the individual data types are missing. For TMAs, The availability of TMA cores stained with CD3 are shown as an example. For most patients who are missing a primary tumor slide, a lymph node slide is available instead. Lymph node WSIs are only available for patients with lymph node metastases.

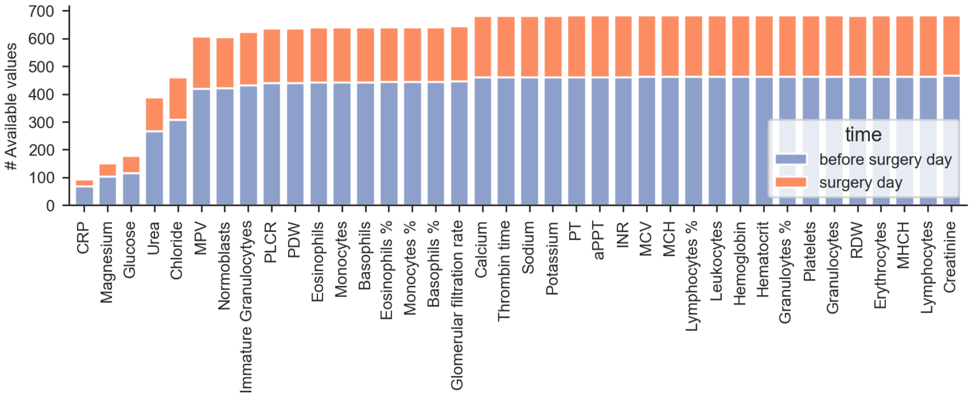

Figure S12 Number of available measurements per parameter, after selecting a single measurement for each parameter and patient. Available pre-operative measurements are highlighted in blue. If this was not available, a measurement from the surgery day (highlighted in red) was selected instead.

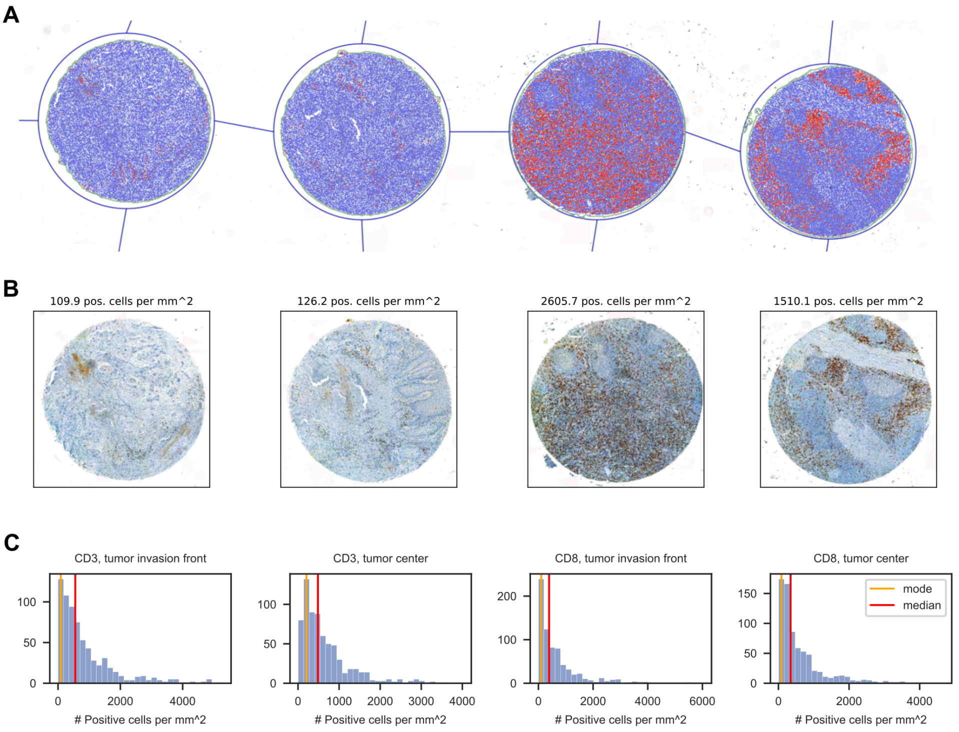

Figure S13 Analysis of cells in Tissue Microarray (TMA) cores. (A) Four exemplary TMA cores with CD8 marker from the tumor invasion front. QuPath was used to de-array the TMA with the core diameter set to 1,9mm. Subsequently, positive cell detection was performed to detect CD8-positive cells. Positive cells are highlighted red and negative cells blue. (B) Number of CD8-positive cells per mm^2 for each core. (C) Histograms showing the distribution of CD3- and CD8-positive cell counts for all patients with available data. The mode and median values are highlighted.

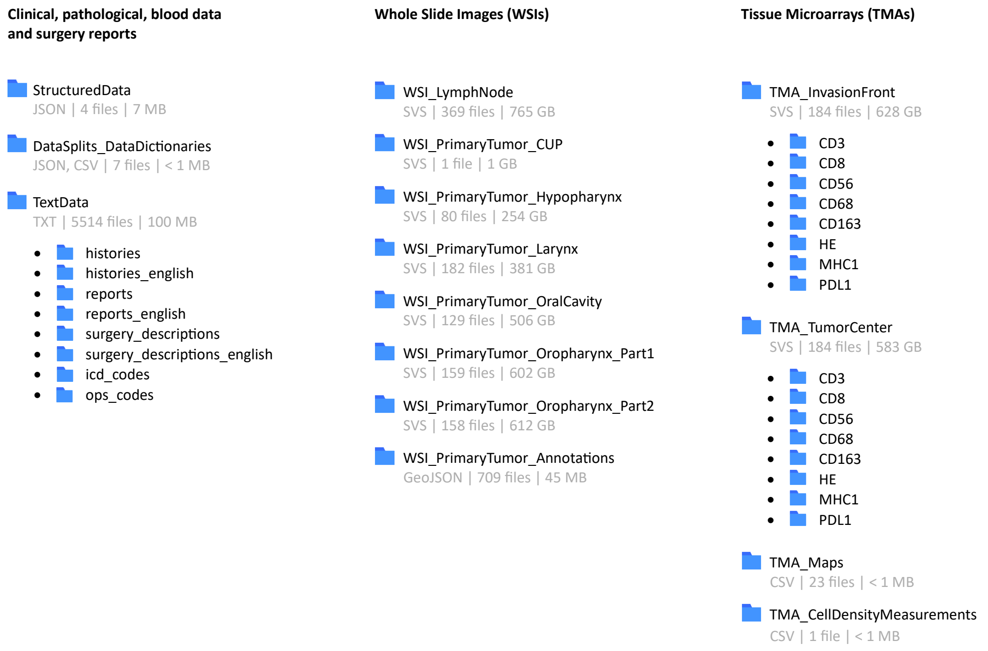

Figure S14 Overview of the dataset, including file formats, numbers of files, and file sizes. The file formats in the dataset are JavaScript Object Notation (JSON), Comma-separated values (CSV), Plain text (TXT), Aperio SVS, and GeoJson. The total size of the dataset is 4.23 terabytes.

Supplementary Tables

Field	Description	Values	Data type
patient_id	Patient identifier, randomly assigned	”001” to ”763”	string
year_of_initial_diagnosis	Year in which the patient was initially diagnosed with cancer		integer
age_at_initial_diagnosis	Patient age in years		integer
sex	Patient sex	female, male	string
smoking_status	First known smoking status of the patient	non-smoker, former, smoker	string
primarily_metastasis	Presence of metastases at the time of diagnosis	no, yes	string
survival_status	Survival status at the time of the last known information	living, deceased	string
survival_status_with_cause	Survival status with cause of death, if documented	living, deceased, deceased not tumor specific, deceased tumor specific	string
days_to_last_information	Time from diagnosis to last information or death, in days		integer
first_treatment_intent	Intent of the first treatment, only patients with curative treatment intent were included	curative	string
first_treatment_modality	Modality of the first treatment, only patients with local surgery were included local surgery		string
first_treatment_descr_german	Short description of the first treatment in German		string
first_treatment_descr_english	Short description of the first treatment, translated to English using ChatGPT-3.5		string
days_to_first_treatment	Time from diagnosis to first treatment, in days, is equal to zero if the diagnosis was confirmed externally		integer
adjuvant_treatment_intent	Intent of adjuvant treatment, if applied	curative, palliative	string
adjuvant_radiotherapy	Use of systemic radiotherapy as adjuvant treatment	no, yes	string
adjuvant_rad_modality	Modality of adjuvant radiotherapy, if applied	brachytherapy, percutaneous radiotherapy	string
adjuvant_systemic_therapy	Use of systemic radiotherapy as adjuvant treatment	no, yes	string
adjuvant_syst_modality	Modality of systemic radiotherapy, if applied	cisplatin, docetaxel, carboplatin, fluorouracil, cetuximab, pembrolizumab	string
adjuvant_rct	Use of radiochemotherapy as adjuvant treatment	no, yes	string
recurrence	Occurrence of a locoregional recurrence of cancer	no, yes	string
days_to_recurrence	Time from diagnosis to recurrence, if any, in days		integer

progress_1	Progress of the cancer, for example distant metastasis, late recurrence, or tumor growth	no, yes	string
days_to_progress_1	Time from diagnosis to first progress, if any, in days		integer
progress_2	Second progress of the cancer, for example distant metastasis, late recurrence, or tumor growth	no, yes	string
days_to_progress_2	Time from diagnosis to second progress, if any, in days		integer
metastasis_1_locations	Localization of first known distant metastases, if present	Adrenal, Bones, Brain, Liver, Lungs, Lymph Nodes, Other Organs, Peritoneum, Pleura, Skin, Soft Tissue, Spleen	string
days_to_metastasis_1	Time from diagnosis to first metastases, if present, in days		integer
metastasis_2_locations	Localization of distant metastases at a second time point, if present	Refer to metastasis_1_locations	string
days_to_metastasis_2	Time from diagnosis to second metastases, if present, in days		integer
metastasis_3_locations	Localization of distant metastases at a third time point, if present	Refer to metastasis_1_locations	string
days_to_metastasis_3	Time from diagnosis to third metastases, if present, in days		integer
metastasis_4_locations	Localization of distant metastases at a fourth time point, if present	Refer to metastasis_1_locations	string
days_to_metastasis_4	Time from diagnosis to fourth metastases, if present, in days		integer

Table S1: Clinical data dictionary.

Field	Description	Values	Data type
patient_id	Patient identifier, randomly assigned	"001" to "763"	string
primary_tumor_site	Site of the primary tumor or CUP (cancer of unknown primary)	Hypopharynx, Larynx, Oral_Cavity, Oropharynx, CUP	string
pT_stage	Pathological T stage, with unknown stage TX	pT1, pT1a, pT1b, pT2, pT3, pT4a, pT4b, pTis, TX	string
pN_stage	Pathological N stage, with unknown stage NX	pN0, pN1, pN1a, pN2, pN2a, pN2b, pN2c, pN3, pN3b, NX	string
grading	Tumor grading, HPV-positive oropharyngeal carcinoma was not graded	G1, G2, G3, HPV_OSCC	string
hpv_association_p16	HPV (human papillomavirus) status as indicated by p16 positivity (=aberrant overexpression), only patients with HPV-positive oropharyngeal carcinoma were tested	negative, positive, not_tested	string
histologic_type	Histologic type of the primary tumor	Mucoepidermoid_Carcinoma, Neuroendocrine_Carcinoma, SCC_Acantholytic, SCC_Basaloid, SCC_Conventional-Keratinizing, SCC_Conventional-NonKeratinizing, SCC_Lymphoepithelial, SCC_Sarcomatoid	string
number_of_positive_lymph_nodes	Number of resected lymph nodes with present cancer cells		integer
number_of_resected_lymph_nodes	Number of resected lymph nodes		integer
perinodal_invasion	Perinodal invasion	no, yes	string
lymphovascular_invasion	Lymphovascular invasion (L)	no, yes	string
vascular_invasion	Vascular invasion (V)	no, yes	string
perineural_invasion_Pn	Perineural invasion (Pn)	no, yes	string
resection_status	Resection status after first treatment, with unknown status RX	R0, R1, R2, RX	string
resection_status_carcinoma_in_situ	Resection status for carcinoma in situ	CIS_absent, Ris0, Ris1	string
carcinoma_in_situ	Presence of carcinoma in situ	no, yes	string
closest_resection_margin_in_cm	Distance to closest resection margin, in cm. Distances smaller than 0.1 cm are denoted with "<0.1"		string
infiltration_depth_in_mm	Infiltration depth of the tumor, in mm		float

Table S2: Pathological data dictionary.

Field	Description	Values	Data type
patient_id	Patient identifier, randomly assigned	"001" to "763"	string
value	Measured value of the analyte		float
unit	Unit	%	string
		fl,	
		g/dl,	
		mg/dl,	
		mg/l,	
		ml/min,	
		mmol/l,	
		pg,	
		s,	
		$\times 10^3/\mu$l	
		$\times 10^6/\mu$l	
analyte_name	Short name of the analyte	Basophils,	string
		Basophils %,	
		CRP,	
		Calcium,	
		Chloride,	
		Creatinine,	
		Eosinophils,	
		Eosinophils %,	
		Erythrocytes,	
		Glomerular filtration rate,	
		Glucose,	
		Granulocytes,	
		Granulocytes %,	
		Hematocrit,	
		Hemoglobin,	
		INR,	
		Immature Granulocytyes,	
		Leukocytes,	
		Lymphocytes,	
		Lymphocytes %,	
		MCH,	
		MCV,	
		MHCH,	
		MPV,	
		Magnesium,	

		Monocytes,	
		Monocytes %,	
		Normoblasts,	
		PDW,	
		PLCR,	
		PT,	
		Platelets,	
		Potassium,	
		RDW,	
		Sodium,	
		Thrombin time,	
		Urea,	
		aPPT	
LOINC_code	LOINC (Logical Observation Identifiers Names and Codes) code	26444-0,	string
		30180-4,	
		1988-5,	
		2000-8,	
		2075-0,	
		2160-0,	
		26449-9,	
		26450-7,	
		26453-1,	
		33914-3,	
		2345-7,	
		30394-1,	
		19023-1,	
		20570-8,	
		718-7,	
		34714-6,	
		38518-7,	
		26464-8,	
		26474-7,	
		26478-8,	
		28539-5,	
		30428-7,	
		28540-3,	
		28542-9,	
		2601-3,	
		26484-6,	
		26485-3,	
		33990-3,	
		32207-3,	

		48386-7,	
		5894-1,	
		26515-7,	
		2823-3,	
		30385-9,	
		2951-2,	
		3243-3,	
		3091-6,	
		3173-2	
LOINC_name	LOINC Long Common Name	Basophils [#/volume] in Blood,	string
		Basophils/100 leukocytes in Blood,	
		C reactive protein [Mass/volume] in Serum or Plasma,	
		Calcium [Moles/volume] in Serum or Plasma,	
		Chloride [Moles/volume] in Serum or Plasma,	
		Creatinine [Mass/volume] in Serum or Plasma,	
		Eosinophils [#/volume] in Blood,	
		Eosinophils/100 leukocytes in Blood,	
		Erythrocytes [#/volume] in Blood,	
		Glomerular filtration rate/1.73 sq M.predicted by Creatinine-based formula (MDRD),	
		Glucose [Mass/volume] in Serum or Plasma,	
		Granulocytes [#/volume] in Blood,	
		Granulocytes/100 leukocytes in Blood by Automated count,	
		Hematocrit [Volume Fraction] of Blood,	
		Hemoglobin [Mass/volume] in Blood,	
		INR in Blood by Coagulation assay,	
		Granulocytes Immature/100 leukocytes in Blood,	
		Leukocytes [#/volume] in Blood,	
		Lymphocytes [#/volume] in Blood,	
		Lymphocytes/100 leukocytes in Blood,	
		Erythrocyte mean corpuscular hemoglobin [Entitic mass],	
		Erythrocyte mean corpuscular volume [Entitic volume],	
		Erythrocyte mean corpuscular hemoglobin concentration [Mass/volume],	
		Platelet mean volume [Entitic volume] in Blood,	
		Magnesium [Moles/volume] in Serum or Plasma,	
		Monocytes [#/volume] in Blood,	
		Monocytes/100 leukocytes in Blood,	
		Normoblasts/100 leukocytes [Ratio] in Blood,	
		Platelet distribution width [Entitic volume] in Blood by Automated count,	
		Platelets Large/Platelets in Blood by Automated count,	

		Prothrombin time (PT) actual/normal in Platelet poor plasma by Coagulation assay,	
		Platelets [#/volume] in Blood,	
		Potassium [Moles/volume] in Serum or Plasma,	
		Erythrocyte distribution width [Ratio],	
		Sodium [Moles/volume] in Serum or Plasma,	
		Thrombin time in Platelet poor plasma by Coagulation assay,	
		Urea [Mass/volume] in Serum or Plasma,	
		Activated partial thromboplastin time (aPTT) in Blood by Coagulation assay	
group	Analyte group	Electrolytes - single valence,	string
		Hematology,	
		Mineral; bone; joint; connective tissue,	
		OG,	
		Protein,	
		Renal function,	
		Routine,	
		Sugars/Sugar metabolism	
days_before_first_treatment	Number of days before the surgery, i.e. 0 corresponds to the surgery day and 1 corresponds to one day before the surgery	[0, 14]	int

Table S3: Blood data dictionary.

Personal identifier	Placeholder	# Replaced in surgery reports	# Replaced in medical histories
Name of a clinician e.g. surgeon	<CLINICIAN_NAME>	941	14
Name of the patient	<PATIENT_NAME>	0	21
Date or month	<[year]>	4	136
Name of a study	<STUDY_NAME>	5	0
Location	<LOCATION>	3	3

Table S4 Number of placeholders used in surgery reports and medical histories for de-identification. Patient names had already been masked in the reports but appeared in some medical histories. Dates in the text were replaced with the corresponding year, for example, <2024>.

Feature Name	Data Type	Encoding Dimensions	$\sum$ Dimensions
sex	binary	1	1
primarily metastasis	binary	1	2
smoking status	categorical	4	6
age at initial diagnosis	numerical	1	7
perinodal invasion	binary	1	8
lymphovascular invasion L	binary	1	9
vascular invasion V	binary	1	10
perineural invasion Pn	binary	1	11
carcinoma in situ	binary	1	12
primary tumor site	categorical	5	17
grading	categorical	4	21
hpv association p16	categorical	3	24
resection status	categorical	4	28
resection status carcinoma in situ	categorical	3	31
histologic type	categorical	9	40
number of positive lymph nodes	numerical	1	41
infiltration depth in mm	numerical	1	42
pT stage	numerical	1	43
pN stage	numerical	1	44
Leukocytes [#/volume] in Blood	numerical	1	45
Hemoglobin [Mass/volume] in Blood	numerical	1	46
Platelets [#/volume] in Blood	numerical	1	47
Erythrocytes [#/volume] in Blood	numerical	1	48
Hematocrit [Volume Fraction] of Blood	numerical	1	49
Erythrocyte mean corpuscular hemoglobin [Entitic mass]	numerical	1	50
Erythrocyte mean corpuscular volume [Entitic volume]	numerical	1	51
Erythrocyte mean corpuscular hemoglobin concentration [Mass/volume]	numerical	1	52
Erythrocyte distribution width [Ratio]	numerical	1	53

Platelet mean volume [Entitic volume] in Blood	numerical	1	54
Granulocytes [#/volume] in Blood	numerical	1	55
Eosinophils [#/volume] in Blood	numerical	1	56
Basophils [#/volume] in Blood	numerical	1	57
Lymphocytes [#/volume] in Blood	numerical	1	58
Monocytes [#/volume] in Blood	numerical	1	59
Platelet distribution width [Entitic volume] in Blood by Automated count	numerical	1	60
ICD-CODE: C0.20	numerical	1	61
ICD-CODE: C0.21	numerical	1	62
ICD-CODE: C0.22	numerical	1	63
ICD-CODE: C0.28	numerical	1	64
ICD-CODE: C0.29	numerical	1	65
ICD-CODE: C0.48	numerical	1	66
ICD-CODE: C0.51	numerical	1	67
ICD-CODE: C0.52	numerical	1	68
ICD-CODE: C0.58	numerical	1	69
ICD-CODE: C0.68	numerical	1	70
ICD-CODE: C0.90	numerical	1	71
ICD-CODE: C0.91	numerical	1	72
ICD-CODE: C0.98	numerical	1	73
ICD-CODE: C0.99	numerical	1	74
ICD-CODE: C1.00	numerical	1	75
ICD-CODE: C1.02	numerical	1	76
ICD-CODE: C1.08	numerical	1	77
ICD-CODE: C1.09	numerical	1	78
ICD-CODE: C1.11	numerical	1	79
ICD-CODE: C1.30	numerical	1	80
ICD-CODE: C1.31	numerical	1	81
ICD-CODE: C1.32	numerical	1	82
ICD-CODE: C1.38	numerical	1	83
ICD-CODE: C1.39	numerical	1	84
ICD-CODE: C1.48	numerical	1	85
ICD-CODE: C3.20	numerical	1	86
ICD-CODE: C3.21	numerical	1	87
ICD-CODE: C3.22	numerical	1	88
ICD-CODE: C3.23	numerical	1	89
ICD-CODE: C3.28	numerical	1	90
ICD-CODE: C3.29	numerical	1	91
ICD-CODE: C7.70	numerical	1	92

ICD-CODE: C7.78	numerical	1	93
ICD-CODE: C8.00	numerical	1	94
ICD-CODE: D0.00	numerical	1	95
ICD-CODE: D3.70	numerical	1	96
ICD-CODE: D3.80	numerical	1	97
ICD-CODE: R5.90	numerical	1	98
ICD-CODE: R5.99	numerical	1	99
ICD-CODE: T8.10	numerical	1	100
cd3 z	numerical	1	101
cd3 inv	numerical	1	102
cd8 z	numerical	1	103
cd8 inv	numerical	1	104

Table S5: Overview of the 104 dimensions of the multimodal feature vector employed for training the various models (see Figure 2). For each patient-specific feature, the table lists the data type and the dimensionality utilized in the encoded feature vector.

Data Split	Model	TPR	FPR	ACC	PPV	REC	SPEC	DICE	AUC
In	RF	0.91	0.01	0.82	0.91	0.30	0.99	0.45	0.80
	AdaBoost	0.59	0.09	0.78	0.59	0.39	0.91	0.47	0.76
	SVC	0.50	0.01	0.75	0.50	0.03	0.99	0.06	0.61
Out	RF	0.79	0.03	0.83	0.79	0.35	0.97	0.49	0.72
	AdaBoost	0.54	0.06	0.77	0.54	0.23	0.94	0.32	0.65
	SVC	nan	0.00	0.77	nan	0.00	1.00	0.00	0.67
Oropharynx	RF	0.61	0.03	0.80	0.61	0.18	0.97	0.28	0.68
	AdaBoost	0.43	0.05	0.78	0.43	0.15	0.95	0.22	0.64
	SVC	0.00	0.00	0.79	0.00	0.00	1.00	0.00	0.55

Table S6 Comparison between a Random Forest (RF), a Support Vector Classifier (SVC) and an AdaBoost model on the task of predicting recurrence. Each model was previously hyperparameter-tuned with 100 iterations of random search on a 10-fold cross-validation with the F1 score as the selection metric. In the ”Out” data split, the SVC did not classify a single instance in the test data set as positive, whereas in the ”Oropharynx” data split, no instance was correctly classified as positive by the SVC. Therefore, not all metrics can be calculated in a valid fashion.
The metrics shown in the table are true positive rate (TPR), false positive rate (FPR), accuracy (ACC), precision (PPV), recall (REC), specificity (SPEC), Dice-Sørensen coefficient (DICE) and receiver operating curve area under the curve (ROC-AUC).

Data Split	Model	TPR	FPR	ACC	PPV	REC	SPEC	DICE	AUC
In	RF	0.40	0.03	0.81	0.40	0.08	0.97	0.13	0.79
	AdaBoost	0.50	0.06	0.82	0.50	0.28	0.94	0.36	0.72
	SVC	0.00	0.02	0.80	0.00	0.00	0.98	0.00	0.62
Out	RF	0.57	0.03	0.81	0.57	0.15	0.97	0.24	0.78
	AdaBoost	0.54	0.05	0.81	0.54	0.26	0.95	0.35	0.70
	SVC	nan	0.00	0.81	nan	0.00	1.00	0.00	0.67
Oropharynx	RF	0.38	0.03	0.81	0.38	0.09	0.97	0.15	0.70
	AdaBoost	0.50	0.03	0.82	0.50	0.15	0.97	0.23	0.65
	SVC	nan	0.00	0.82	nan	0.00	1.00	0.00	0.52

Table S7 Comparison between a Random Forest (RF), a Support Vector Classifier (SVC) and an Ada Boost model on the task of predicting survival status. Each model was previously hyperparameter-tuned with 100 iterations of random search on a 10-fold cross-validation with the F1 score as the selection metric. The SVC does not predict a single instance in the test set as positive in the ”Oropharynx” and ”Out” data splits. Therefore, not all metrics can be calculated in a valid fashion.
The metrics shown in the table are true positive rate (TPR), false positive rate (FPR), accuracy (ACC), precision (PPV), recall (REC), specificity (SPEC), Dice-Sørensen coefficient (DICE) and receiver operating curve area under the curve (ROC-AUC).
