# Supplementary material for: A multimodal dataset for precision oncology in head and neck cancer
Source: Nat Commun. 2025 Aug 4;16:7163. doi: 10.1038/s41467-025-62386-6 (PMC12322140; doi:10.1038/s41467-025-62386-6)
Supplement: Supplementary file 3 — Reporting Summary [file 41467_2025_62386_MOESM3_ESM.pdf]

nature portfolio

Corresponding author(s): Andreas Kist

Last updated by author(s): May 31, 2024

Reporting Summary

Nature Portfolio wishes to improve the reproducibility of the work that we publish. This form provides structure for consistency and transparency in reporting. For further information on Nature Portfolio policies, see our Editorial Policies and the Editorial Policy Checklist.

Statistics

For all statistical analyses, confirm that the following items are present in the figure legend, table legend, main text, or Methods section.

n/a	Confirmed	
☐	☒	The exact sample size (*n*) for each experimental group/condition, given as a discrete number and unit of measurement
☐	☒	A statement on whether measurements were taken from distinct samples or whether the same sample was measured repeatedly
☐	☒	The statistical test(s) used AND whether they are one- or two-sided<br>*Only common tests should be described solely by name; describe more complex techniques in the Methods section.*
☒	☐	A description of all covariates tested
☐	☒	A description of any assumptions or corrections, such as tests of normality and adjustment for multiple comparisons
☐	☒	A full description of the statistical parameters including central tendency (e.g. means) or other basic estimates (e.g. regression coefficient) AND variation (e.g. standard deviation) or associated estimates of uncertainty (e.g. confidence intervals)
☒	☐	For null hypothesis testing, the test statistic (e.g. *F*, *t*, *r*) with confidence intervals, effect sizes, degrees of freedom and *P* value noted<br>*Give P values as exact values whenever suitable.*
☒	☐	For Bayesian analysis, information on the choice of priors and Markov chain Monte Carlo settings
☒	☐	For hierarchical and complex designs, identification of the appropriate level for tests and full reporting of outcomes
☒	☐	Estimates of effect sizes (e.g. Cohen's *d*, Pearson's *r*), indicating how they were calculated

Our web collection on statistics for biologists contains articles on many of the points above.

Software and code

Policy information about availability of computer code

Data collection	Commercial Scanners and their software (3DHistech P1000, Biosystems GT450), QuPath, Python 3.9 and later
Data analysis	Python and related libraries (pandas 2.2.3, numpy 2.0.2, TensorFlow 2.18); https://github.com/ankilab/HANCOCK_MultimodalDataset

For manuscripts utilizing custom algorithms or software that are central to the research but not yet described in published literature, software must be made available to editors and reviewers. We strongly encourage code deposition in a community repository (e.g. GitHub). See the Nature Portfolio guidelines for submitting code & software for further information.

Data

Policy information about availability of data

All manuscripts must include a data availability statement. This statement should provide the following information, where applicable:

- Accession codes, unique identifiers, or web links for publicly available datasets
- A description of any restrictions on data availability
- For clinical datasets or third party data, please ensure that the statement adheres to our policy

The HANCOCK dataset is publicly available at https://hancock.research.fau.eu/ and will be mirrored to The Cancer Imaging Archive (TCIA) at https://doi.org/10.7937/rcty-5h16. An overview of the dataset, including the number and format of files, is shown in Supplementary Fig. S14. The underlying data plotted in the main Figures 1-3 is available in the Source Data file.

Research involving human participants, their data, or biological material

Policy information about studies with human participants or human data. See also policy information about sex, gender (identity/presentation), and sexual orientation and race, ethnicity and racism.

Reporting on sex and gender	Retrospective data was collected at the ENT department of the University Hospital Erlangen. In total, 763 patients were included. 80% identified themselves as men. The relative frequency reflects the typical prevalence of head& neck cancer (majority suffering from head&neck are men). Gender was determined based on self-report. Gender had no role in study design.
Reporting on race, ethnicity, or other socially relevant groupings	The data stems from a clinic in Germany. We expect the typical demographics in Germany. Race, ethnicity, or other socially relevant groupings have not been considered or controlled for.
Population characteristics	We provide head and neck cancer data, the median age is 61, with the lowest age reported is 22 and the highest is 92 at time of diagnosis. Details are given in the paper.
Recruitment	N/A (retrospective study)
Ethics oversight	Ethics board - Medical Faculty - Friedrich-Alexander-University Erlangen-Nürnberg, informed consent was waived.

Note that full information on the approval of the study protocol must also be provided in the manuscript.

Field-specific reporting

Please select the one below that is the best fit for your research. If you are not sure, read the appropriate sections before making your selection.

☒ Life sciences ☐ Behavioural & social sciences ☐ Ecological, evolutionary & environmental sciences

For a reference copy of the document with all sections, see nature.com/documents/nr-reporting-summary-flat.pdf

Life sciences study design

All studies must disclose on these points even when the disclosure is negative.

Sample size	All available samples were included.
Data exclusions	Subjects lacking data modalities were excluded from comparisons relying on this given modality.
Replication	We performed nested k-fold crossvalidation and train/validation/test splits wherever applicable. Details are in the paper.
Randomization	We randomized the patients in the train/validation/test splits and nested k-fold crossvalidations wherever applicable.
Blinding	For machine and deep learning analyses, no blinding is necessary.

Reporting for specific materials, systems and methods

We require information from authors about some types of materials, experimental systems and methods used in many studies. Here, indicate whether each material, system or method listed is relevant to your study. If you are not sure if a list item applies to your research, read the appropriate section before selecting a response.

Materials & experimental systems

n/a	Involved in the study	
☒	☐	Antibodies
☒	☐	Eukaryotic cell lines
☒	☐	Palaeontology and archaeology
☒	☐	Animals and other organisms
☒	☐	Clinical data
☒	☐	Dual use research of concern
☒	☐	Plants

Methods

n/a	Involved in the study	
☒	☐	ChIP-seq
☒	☐	Flow cytometry
☒	☐	MRI-based neuroimaging

Plants

Seed stocks	*Report on the source of all seed stocks or other plant material used. If applicable, state the seed stock centre and catalogue number. If plant specimens were collected from the field, describe the collection location, date and sampling procedures.*
Novel plant genotypes	*Describe the methods by which all novel plant genotypes were produced. This includes those generated by transgenic approaches, gene editing, chemical/radiation-based mutagenesis and hybridization. For transgenic lines, describe the transformation method, the number of independent lines analyzed and the generation upon which experiments were performed. For gene-edited lines, describe the editor used, the endogenous sequence targeted for editing, the targeting guide RNA sequence (if applicable) and how the editor was applied.*
Authentication	*Describe any authentication procedures for each seed stock used or novel genotype generated. Describe any experiments used to assess the effect of a mutation and, where applicable, how potential secondary effects (e.g. second site T-DNA insertions, mosiacism, off-target gene editing) were examined.*
